# *N*-(9,11-Dimeth­oxy-4-oxo-2,3,4,6,7,11b-hexa­hydro-1*H*-pyrido[2,1-*a*]isoquinolin-3-yl)benzamide

**DOI:** 10.1107/S1600536810050737

**Published:** 2010-12-11

**Authors:** Hoong-Kun Fun, Punlop Kuntiyong, Pittaya Tuntiwachwuttikul, Suchada Chantrapromma

**Affiliations:** a X-ray Crystallography Unit, School of Physics, Universiti Sains Malaysia, 11800 USM, Penang, Malaysia; b Department of Chemistry, Faculty of Science, Silpakorn University, Rajamanka Nai Road, Muang Nakhon Pathom 73000, Thailand; c Crystal Materials Research Unit, Department of Chemistry, Faculty of Science, Prince of Songkla University, Hat-Yai, Songkhla 90112, Thailand

## Abstract

The title schulzeine derivative, C_22_H_24_N_2_O_4_, crystallizes with two crystallographically independent mol­ecules of almost identical conformation in the asymmetric unit. The tricyclic core of schulzeine has a fused-three-ring system comprising the tetra­hydro­isoquinoline and δ-lactam moieties. In both mol­ecules, the pyridine ring adopts a twisted-boat conformation, whereas the lactam ring is in a boat conformation. The two meth­oxy groups are slightly twisted from the attached benzene ring [C—O—C—C torsion angles = −21.3 (2) and −20.5 (2)° in mol­ecule *A*, and −6.3 (2) and −16.2 (2)° in mol­ecule *B*] and the benzamide moiety is in a (−)-synclinal conformation with respect to the lactam ring. In the crystal, mol­ecules are linked into V-shaped dimers by inter­molecular N—H⋯O hydrogen bonds and weak C—H⋯O inter­actions. These dimers are stacked into V-shaped columns along the *a* axis. Adjacent columns are further linked in an anti­parallel manner. C—H⋯π inter­actions are also observed.

## Related literature

For hydrogen-bond motifs, see: Bernstein *et al.* (1995 ▶). For bond-length data, see: Allen *et al.* (1987 ▶). For ring conformations, see: Cremer & Pople (1975 ▶). For background to schulzeines, see, for example: Kuntiyong *et al.* (2006 ▶); Melo *et al.* (2006 ▶); Takada *et al.* (2004) ▶. For the stability of the temperature controller used in the data collection, see: Cosier & Glazer (1986) ▶.
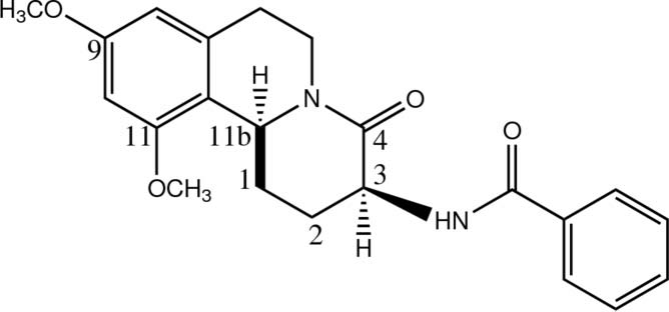


## Experimental

### 

#### Crystal data

C_22_H_24_N_2_O_4_
*M* *_r_* = 380.43
Orthorhombic, 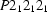

*a* = 12.6530 (3) Å
*b* = 15.5256 (4) Å
*c* = 19.7819 (5) Å
*V* = 3886.06 (17) Å^3^
*Z* = 8
Mo *K*α radiation
μ = 0.09 mm^−1^
*T* = 100 K
0.56 × 0.44 × 0.41 mm

#### Data collection

Bruker SMART APEX2 CCD area-detector diffractometer
Absorption correction: multi-scan (*SADABS*; Bruker, 2005 ▶) *T*_min_ = 0.952, *T*_max_ = 0.964
110999 measured reflections
6224 independent reflections
5979 reflections with *I* > 2σ(*I*)
*R*_int_ = 0.044

#### Refinement

*R*[*F* ^2^ > 2σ(*F* ^2^)] = 0.037
*wR*(*F* ^2^) = 0.098
*S* = 1.04
6224 reflections
509 parameters
H-atom parameters constrained
Δρ_max_ = 0.60 e Å^−3^
Δρ_min_ = −0.21 e Å^−3^

### 

Data collection: *APEX2* (Bruker, 2005 ▶); cell refinement: *SAINT* (Bruker, 2005 ▶); data reduction: *SAINT*; program(s) used to solve structure: *SHELXTL* (Sheldrick, 2008 ▶); program(s) used to refine structure: *SHELXTL*; molecular graphics: *SHELXTL*; software used to prepare material for publication: *SHELXTL* and *PLATON* (Spek, 2009 ▶).

## Supplementary Material

Crystal structure: contains datablocks global, I. DOI: 10.1107/S1600536810050737/rz2531sup1.cif

Structure factors: contains datablocks I. DOI: 10.1107/S1600536810050737/rz2531Isup2.hkl

Additional supplementary materials:  crystallographic information; 3D view; checkCIF report

## Figures and Tables

**Table 1:** Hydrogen-bond geometry (Å, °) *Cg*1 and *Cg*2 are the centroids of the C1*A*–C6*A* and C15*A*–C20*A* benzene rings, respectively.

*D*—H⋯*A*	*D*—H	H⋯*A*	*D*⋯*A*	*D*—H⋯*A*
N2*A*—H2*AA*⋯O1*B*^i^	0.86	2.08	2.8994 (18)	158
N2*B*—H2*BA*⋯O1*A*^ii^	0.86	2.24	2.9807 (18)	145
C1*A*—H1*AA*⋯O4*B*^iii^	0.93	2.52	3.405 (2)	160
C10*A*—H10*B*⋯O3*A*	0.97	2.58	3.127 (2)	115
C3*B*—H3*BA*⋯O4*A*^iv^	0.93	2.49	3.348 (2)	153
C7*B*—H7*BB*⋯O4*A*^v^	0.97	2.47	3.407 (2)	163
C16*A*—H16*A*⋯O1*B*^i^	0.93	2.32	3.228 (2)	165
C20*B*—H20*B*⋯O1*A*^ii^	0.93	2.51	3.281 (2)	141
C22*A*—H22*A*⋯O2*A*^iv^	0.96	2.52	3.266 (2)	135
C22*A*—H22*B*⋯O4*B*^vi^	0.96	2.47	3.393 (2)	160
C22*B*—H22*D*⋯O4*A*^iv^	0.96	2.58	3.414 (2)	146
C11*A*—H11*B*⋯*Cg*2^ii^	0.97	2.78	3.686 (2)	156
C8*B*—H8*BA*⋯*Cg*1^iv^	0.97	2.60	3.4864 (17)	152
C18*B*—H18*B*⋯*Cg*1^i^	0.93	2.81	3.607 (2)	144

Symmetry codes: (i) 
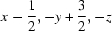
; (ii) 
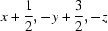
; (iii) 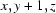; (iv) 
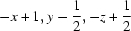
; (v) 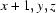; (vi) 
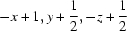
.

**Fig. 1.**
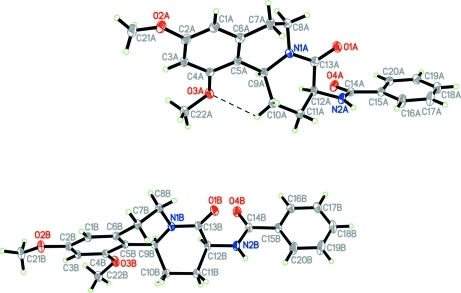
The molecular structure of the title compound, showing 50% probability displacement ellipsoids and the atomic numbering. The intramolecular C—H···O weak interaction is shown as dash line.

**Fig. 2.**
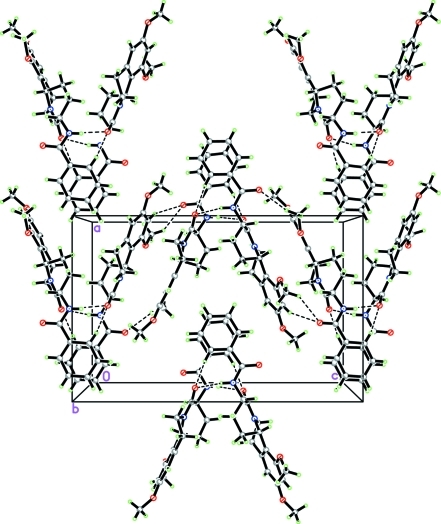
The crystal packing of the title compound, viewed down the b axis. Hydrogen bonds are shown as dashed lines.

## supplementary crystallographic information

### Comment

Schulzeine alkaloids were isolated from the marine sponge *Penares schulzei* (Takada *et al.*, 2004). Schulzenine A—C inhibits α-glucosidase with IC_50_ values of 48–170 nM (Melo *et al.*, 2006). The potent α-glucosidase inhibitory activity makes them attractive leads for drug candidate for diseases such as cancer, diabetes and viral infections. During the course of our research on natural product synthesis of schulzeines, the title schulzeine benzamide derivative was synthesized (Kuntiyong *et al.*, 2006). The single-crystal *x*-ray structural study of the title compound (I) was undertaken in order to gain detailed conformations of the molecular structure.

The title compound crystallized with two crystallographically independent molecues *A* and *B* in the asymmetric unit (Fig. 1). These two molecules are almost conformationally identical with slightly different bond lengths and angles. The bond lengths in (I) are within normal ranges (Allen *et al.*, 1987). The tricyclic core of schulzeine (C1–C13/N1/O1) has a fused-three-ring system comprising of the tetrahydro isoquinoline and δ-lactam moieties. In both molecules, the pyridine ring (C5–C9/N1) adopts a twisted boat conformation with puckering parameters Q = 0.6943 (17) Å, θ = 92.19 (14)° and φ = 245.21 (14)° in molecule *A* [Q = 0.5265 (16) Å, θ = 112.63 (17)° and φ = 263.90 (19)° in molecule *B*] and the lactam ring (C9–C13/N1/O1) has a standard boat conformation (Cremer & Pople 1975). The two methoxy groups are slightly twisted from the attached benzene ring with the torsion angles C21–O2–C2–C3 = -21.3 (2)° and C22–O3–C4–C3 = -20.5 (2)° in molecule *A* [the corresponding values are -6.3 (2) and -16.2 (2)° in molecule *B*]. The benzamide moiety is not planar with the dihedral angle between the mean plane through C14–C15/N2/O4 and C15–C20 benzene ring being 8.97 (9)° in molecule *A* [8.10 (11)° in molecule *B*]. The orientation of the benzamide moiety can be described by the torsion angle C14–N2–C12–C13 = -92.77 (17)° in molecule *A* [-82.56 (18)° in molecule *B*] which shows the (-)-*syn*-clinal conformation with respect to the lactam ring. There are two stereogenic centers at atoms C12 and C9 (Fig. 1) or postions 3 and 11*b* of the tricyclic core of schulzeine. Fig. 1 show that H atoms at C12 and C9 are in *cis*-relationship. An intramolecular C10A—H10B···O3A weak interaction (Table 1; Fig. 1) generates a S(6) ring motif (Bernstein *et al.*, 1995).

In the crystal packing (Fig. 2), molecules are linked into V-shaped dimers by N—H···O hydrogen bonds and C—H···O weak interactions (Table 1) which generate two S(7) ring motifs (Bernstein *et al.*, 1995). These dimers are stacked into V-shaped columns along the *a* axis. Adjacent columns are further linked by C—H···O weak interactions in an antiparallel manner. C—H···π interactions were also observed (Table 1); *Cg*_1_ and *Cg*_2_ are the centroid of C1A–C6A and C15A–C20A benzene rings, respectively.

### Experimental

The title schulzeine benzamide derivative was synthesized according to the previous reported method (Kuntiyong *et al.*, 2006). Colourless block-shaped single crystals of the title compound suitable for *x*-ray structure determination were crystallized from hexane:ethylacetate (2:1 *v*/*v*) by slow evaporation at room temperature after a few days.

### Refinement

All H atoms were placed in calculated positions with d(N—H) = 0.86 Å, d(C—H) = 0.93 Å for aromatic, 0.97 for CH_2_ and 0.96 Å for CH_3_ atoms. The *U*_iso_ values were constrained to be 1.5*U*_eq_ of the carrier atom for methyl H atoms and 1.2*U*_eq_ for the remaining H atoms. A rotating group model was used for the methyl groups. The highest residual electron density peak is located at 2.33 Å from H21G and the deepest hole is located at 1.38 Å from C5B. A total of 5097 Friedel pairs were merged before final refinement as there is no large anomalous dispersion for the determination of the absolute configuration.

### Crystal data

**Table:**

C_22_H_24_N_2_O_4_	*F*(000) = 1616
*M**_r_* = 380.43	*D*_x_ = 1.301 Mg m^−^^3^
Orthorhombic, *P*2_1_2_1_2_1_	Mo *K*α radiation, λ = 0.71073 Å
Hall symbol: P 2ac 2ab	Cell parameters from 6224 reflections
*a* = 12.6530 (3) Å	θ = 1.7–30.0°
*b* = 15.5256 (4) Å	µ = 0.09 mm^−^^1^
*c* = 19.7819 (5) Å	*T* = 100 K
*V* = 3886.06 (17) Å^3^	Block, colourless
*Z* = 8	0.56 × 0.44 × 0.41 mm

### Data collection

**Table:**

Bruker SMART APEX2 CCD area-detector diffractometer	6224 independent reflections
Radiation source: fine-focus sealed tube	5979 reflections with *I* > 2σ(*I*)
graphite	*R*_int_ = 0.044
Detector resolution: 8.33 pixels mm^-1^	θ_max_ = 30.0°, θ_min_ = 1.7°
ω scans	*h* = −17→17
Absorption correction: multi-scan (*SADABS*; Bruker, 2005)	*k* = −21→21
*T*_min_ = 0.952, *T*_max_ = 0.964	*l* = −27→27
110999 measured reflections	

### Refinement

**Table:**

Refinement on *F*^2^	Primary atom site location: structure-invariant direct methods
Least-squares matrix: full	Secondary atom site location: difference Fourier map
*R*[*F*^2^ > 2σ(*F*^2^)] = 0.037	Hydrogen site location: inferred from neighbouring sites
*wR*(*F*^2^) = 0.098	H-atom parameters constrained
*S* = 1.04	*w* = 1/[σ^2^(*F*_o_^2^) + (0.0603*P*)^2^ + 0.8464*P*] where *P* = (*F*_o_^2^ + 2*F*_c_^2^)/3
6224 reflections	(Δ/σ)_max_ = 0.001
509 parameters	Δρ_max_ = 0.60 e Å^−^^3^
0 restraints	Δρ_min_ = −0.21 e Å^−^^3^

### Special details

**Table:**

Experimental. The low-temparture data was collected with the Oxford Cyrosystem Cobra low-temperature attachment.
Geometry. All e.s.d.'s (except the e.s.d. in the dihedral angle between two l.s. planes) are estimated using the full covariance matrix. The cell e.s.d.'s are taken into account individually in the estimation of e.s.d.'s in distances, angles and torsion angles; correlations between e.s.d.'s in cell parameters are only used when they are defined by crystal symmetry. An approximate (isotropic) treatment of cell e.s.d.'s is used for estimating e.s.d.'s involving l.s. planes.
Refinement. Refinement of *F*^2^ against ALL reflections. The weighted *R*-factor *wR* and goodness of fit *S* are based on *F*^2^, conventional *R*-factors *R* are based on *F*, with *F* set to zero for negative *F*^2^. The threshold expression of *F*^2^ > σ(*F*^2^) is used only for calculating *R*-factors(gt) *etc*. and is not relevant to the choice of reflections for refinement. *R*-factors based on *F*^2^ are statistically about twice as large as those based on *F*, and *R*- factors based on ALL data will be even larger.

### Fractional atomic coordinates and isotropic or equivalent isotropic displacement parameters (Å^2^)

**Table:**

	*x*	*y*	*z*	*U*_iso_*/*U*_eq_	
O1A	−0.02471 (9)	1.04124 (8)	0.08332 (6)	0.0221 (2)	
O2A	0.57498 (10)	1.20708 (8)	0.23122 (7)	0.0271 (3)	
O3A	0.41894 (9)	0.92533 (8)	0.19545 (6)	0.0199 (2)	
O4A	−0.09182 (10)	0.81182 (8)	0.13037 (6)	0.0216 (2)	
N1A	0.14296 (10)	1.04299 (9)	0.12475 (7)	0.0175 (2)	
N2A	−0.01509 (11)	0.87770 (10)	0.04098 (7)	0.0187 (3)	
H2AA	−0.0159	0.8832	−0.0023	0.022*	
C1A	0.41044 (14)	1.19103 (11)	0.17940 (8)	0.0213 (3)	
H1AA	0.4082	1.2506	0.1745	0.026*	
C2A	0.49678 (13)	1.15230 (11)	0.20998 (8)	0.0206 (3)	
C3A	0.50218 (12)	1.06320 (11)	0.21678 (8)	0.0187 (3)	
H3AA	0.5600	1.0373	0.2375	0.022*	
C4A	0.41891 (12)	1.01348 (10)	0.19180 (8)	0.0166 (3)	
C5A	0.32962 (12)	1.05092 (10)	0.16217 (7)	0.0163 (3)	
C6A	0.32713 (13)	1.14081 (11)	0.15601 (8)	0.0187 (3)	
C7A	0.23051 (14)	1.18307 (11)	0.12658 (9)	0.0222 (3)	
H7AA	0.2361	1.1848	0.0777	0.027*	
H7AB	0.2250	1.2418	0.1430	0.027*	
C8A	0.13342 (13)	1.13264 (11)	0.14688 (9)	0.0209 (3)	
H8AA	0.1252	1.1346	0.1956	0.025*	
H8AB	0.0712	1.1584	0.1266	0.025*	
C9A	0.24112 (12)	0.99366 (10)	0.13682 (7)	0.0158 (3)	
H9AA	0.2268	0.9495	0.1710	0.019*	
C10A	0.27105 (13)	0.94854 (11)	0.07015 (8)	0.0189 (3)	
H10A	0.2925	0.9916	0.0374	0.023*	
H10B	0.3309	0.9108	0.0781	0.023*	
C11A	0.17958 (13)	0.89550 (12)	0.04059 (8)	0.0212 (3)	
H11A	0.1696	0.9114	−0.0064	0.025*	
H11B	0.1982	0.8349	0.0420	0.025*	
C12A	0.07537 (12)	0.90884 (11)	0.07883 (8)	0.0170 (3)	
H12A	0.0792	0.8766	0.1213	0.020*	
C13A	0.05917 (12)	1.00405 (11)	0.09583 (7)	0.0174 (3)	
C14A	−0.09814 (12)	0.84027 (10)	0.07226 (8)	0.0166 (3)	
C15A	−0.20086 (12)	0.83599 (10)	0.03375 (8)	0.0166 (3)	
C16A	−0.21118 (14)	0.86011 (13)	−0.03395 (9)	0.0257 (4)	
H16A	−0.1519	0.8769	−0.0584	0.031*	
C17A	−0.30990 (15)	0.85908 (14)	−0.06489 (9)	0.0292 (4)	
H17A	−0.3163	0.8755	−0.1099	0.035*	
C18A	−0.39848 (14)	0.83383 (12)	−0.02912 (9)	0.0249 (3)	
H18A	−0.4642	0.8330	−0.0501	0.030*	
C19A	−0.38905 (13)	0.80972 (12)	0.03818 (9)	0.0224 (3)	
H19A	−0.4486	0.7927	0.0623	0.027*	
C20A	−0.29063 (13)	0.81098 (10)	0.06949 (8)	0.0186 (3)	
H20A	−0.2847	0.7950	0.1146	0.022*	
C21A	0.64678 (16)	1.17622 (14)	0.28122 (12)	0.0334 (4)	
H21A	0.6889	1.2232	0.2976	0.050*	
H21B	0.6920	1.1333	0.2617	0.050*	
H21C	0.6078	1.1514	0.3180	0.050*	
C22A	0.51984 (13)	0.88467 (11)	0.20440 (9)	0.0223 (3)	
H22A	0.5121	0.8234	0.1998	0.033*	
H22B	0.5466	0.8978	0.2486	0.033*	
H22C	0.5682	0.9056	0.1708	0.033*	
O1B	0.50160 (9)	0.55662 (8)	0.09581 (6)	0.0203 (2)	
O2B	1.13487 (9)	0.53207 (8)	0.24302 (6)	0.0230 (3)	
O3B	0.85002 (9)	0.32332 (7)	0.22556 (6)	0.0209 (2)	
O4B	0.33646 (10)	0.39884 (9)	0.15123 (6)	0.0241 (3)	
N1B	0.65877 (11)	0.50975 (8)	0.13713 (7)	0.0159 (2)	
N2B	0.43821 (11)	0.39672 (10)	0.05729 (7)	0.0193 (3)	
H2BA	0.4403	0.3913	0.0140	0.023*	
C1B	0.97678 (12)	0.56373 (10)	0.18722 (8)	0.0167 (3)	
H1BA	1.0046	0.6177	0.1773	0.020*	
C2B	1.03665 (12)	0.50468 (11)	0.22370 (8)	0.0174 (3)	
C3B	0.99674 (12)	0.42308 (10)	0.23801 (8)	0.0170 (3)	
H3BA	1.0365	0.3834	0.2624	0.020*	
C4B	0.89549 (12)	0.40221 (10)	0.21479 (7)	0.0153 (3)	
C5B	0.83320 (12)	0.46120 (10)	0.17890 (7)	0.0150 (3)	
C6B	0.87571 (12)	0.54239 (10)	0.16559 (7)	0.0153 (3)	
C7B	0.80872 (12)	0.60788 (10)	0.12956 (8)	0.0176 (3)	
H7BA	0.8141	0.5997	0.0811	0.021*	
H7BB	0.8332	0.6655	0.1402	0.021*	
C8B	0.69425 (12)	0.59717 (10)	0.15200 (8)	0.0171 (3)	
H8BA	0.6885	0.6081	0.2001	0.021*	
H8BB	0.6499	0.6383	0.1284	0.021*	
C9B	0.72515 (11)	0.43407 (10)	0.15402 (8)	0.0146 (3)	
H9BA	0.6901	0.4013	0.1899	0.018*	
C10B	0.73405 (13)	0.37627 (11)	0.09087 (8)	0.0198 (3)	
H10C	0.7815	0.4032	0.0587	0.024*	
H10D	0.7645	0.3214	0.1038	0.024*	
C11B	0.62681 (13)	0.36037 (12)	0.05659 (9)	0.0222 (3)	
H11C	0.6290	0.3829	0.0109	0.027*	
H11D	0.6142	0.2988	0.0539	0.027*	
C12B	0.53572 (12)	0.40287 (11)	0.09492 (8)	0.0168 (3)	
H12B	0.5265	0.3727	0.1380	0.020*	
C13B	0.56258 (12)	0.49707 (10)	0.10982 (7)	0.0161 (3)	
C14B	0.34367 (12)	0.39942 (10)	0.08899 (8)	0.0177 (3)	
C15B	0.24663 (12)	0.39956 (11)	0.04551 (8)	0.0191 (3)	
C16B	0.15036 (14)	0.41091 (14)	0.07706 (10)	0.0297 (4)	
H16B	0.1480	0.4180	0.1237	0.036*	
C17B	0.05695 (15)	0.41197 (17)	0.04015 (12)	0.0377 (5)	
H17B	−0.0073	0.4199	0.0621	0.045*	
C18B	0.05931 (15)	0.40119 (15)	−0.02913 (11)	0.0331 (4)	
H18B	−0.0030	0.4028	−0.0540	0.040*	
C19B	0.15575 (16)	0.38786 (17)	−0.06142 (10)	0.0381 (5)	
H19B	0.1576	0.3793	−0.1079	0.046*	
C20B	0.24924 (15)	0.38723 (16)	−0.02456 (9)	0.0316 (4)	
H20B	0.3134	0.3786	−0.0464	0.038*	
C21B	1.19478 (14)	0.47576 (13)	0.28599 (10)	0.0257 (4)	
H21D	1.2585	0.5042	0.3000	0.038*	
H21G	1.2124	0.4242	0.2617	0.038*	
H21E	1.1535	0.4612	0.3251	0.038*	
C22B	0.91795 (15)	0.25465 (11)	0.24549 (10)	0.0246 (3)	
H22G	0.8806	0.2010	0.2422	0.037*	
H22D	0.9404	0.2635	0.2913	0.037*	
H22E	0.9786	0.2532	0.2164	0.037*	

### Atomic displacement parameters (Å^2^)

**Table:**

	*U*^11^	*U*^22^	*U*^33^	*U*^12^	*U*^13^	*U*^23^
O1A	0.0152 (5)	0.0286 (6)	0.0224 (5)	0.0016 (5)	−0.0013 (4)	0.0001 (5)
O2A	0.0211 (6)	0.0247 (6)	0.0356 (7)	−0.0071 (5)	−0.0059 (5)	−0.0060 (5)
O3A	0.0150 (5)	0.0189 (5)	0.0257 (6)	−0.0001 (4)	−0.0024 (4)	0.0005 (4)
O4A	0.0216 (6)	0.0264 (6)	0.0167 (5)	−0.0076 (5)	−0.0026 (4)	0.0036 (4)
N1A	0.0135 (6)	0.0196 (6)	0.0193 (6)	0.0008 (5)	−0.0005 (5)	−0.0012 (5)
N2A	0.0147 (6)	0.0260 (7)	0.0153 (6)	−0.0054 (5)	−0.0024 (5)	0.0021 (5)
C1A	0.0227 (8)	0.0187 (7)	0.0227 (7)	−0.0042 (6)	0.0001 (6)	−0.0014 (6)
C2A	0.0166 (7)	0.0242 (8)	0.0209 (7)	−0.0050 (6)	0.0015 (6)	−0.0054 (6)
C3A	0.0138 (7)	0.0232 (7)	0.0192 (7)	−0.0020 (6)	−0.0001 (6)	−0.0026 (6)
C4A	0.0145 (7)	0.0186 (7)	0.0168 (6)	−0.0009 (5)	0.0017 (5)	−0.0019 (5)
C5A	0.0143 (6)	0.0188 (7)	0.0157 (6)	−0.0027 (6)	0.0006 (5)	−0.0015 (5)
C6A	0.0187 (7)	0.0205 (7)	0.0169 (6)	−0.0022 (6)	0.0000 (6)	0.0007 (6)
C7A	0.0224 (8)	0.0189 (7)	0.0254 (7)	−0.0007 (6)	−0.0051 (7)	0.0026 (6)
C8A	0.0181 (7)	0.0207 (7)	0.0239 (7)	0.0029 (6)	−0.0019 (6)	−0.0024 (6)
C9A	0.0129 (6)	0.0182 (6)	0.0162 (6)	−0.0016 (5)	−0.0005 (5)	0.0002 (5)
C10A	0.0150 (7)	0.0236 (7)	0.0181 (6)	−0.0032 (6)	0.0010 (5)	−0.0032 (6)
C11A	0.0145 (7)	0.0254 (8)	0.0235 (7)	−0.0012 (6)	−0.0011 (6)	−0.0069 (6)
C12A	0.0127 (6)	0.0223 (7)	0.0159 (6)	−0.0036 (6)	−0.0013 (5)	0.0013 (5)
C13A	0.0150 (6)	0.0243 (7)	0.0128 (6)	−0.0024 (6)	0.0020 (5)	0.0016 (5)
C14A	0.0153 (6)	0.0169 (6)	0.0176 (6)	−0.0017 (6)	−0.0009 (5)	−0.0014 (5)
C15A	0.0143 (6)	0.0174 (7)	0.0180 (6)	−0.0029 (5)	−0.0007 (5)	0.0002 (5)
C16A	0.0186 (8)	0.0366 (10)	0.0220 (8)	−0.0094 (7)	−0.0027 (6)	0.0058 (7)
C17A	0.0228 (8)	0.0416 (10)	0.0233 (8)	−0.0095 (8)	−0.0072 (7)	0.0087 (7)
C18A	0.0166 (7)	0.0294 (8)	0.0287 (8)	−0.0024 (7)	−0.0061 (6)	0.0012 (7)
C19A	0.0149 (7)	0.0277 (8)	0.0248 (8)	−0.0016 (6)	0.0018 (6)	−0.0008 (7)
C20A	0.0165 (7)	0.0195 (7)	0.0196 (7)	−0.0015 (6)	0.0013 (6)	−0.0005 (6)
C21A	0.0227 (9)	0.0336 (10)	0.0440 (11)	−0.0034 (8)	−0.0113 (8)	−0.0088 (9)
C22A	0.0172 (7)	0.0243 (8)	0.0254 (8)	0.0022 (6)	−0.0036 (6)	0.0025 (6)
O1B	0.0162 (5)	0.0241 (6)	0.0207 (5)	0.0038 (5)	−0.0023 (4)	0.0040 (4)
O2B	0.0140 (5)	0.0276 (6)	0.0273 (6)	−0.0045 (5)	−0.0055 (5)	0.0033 (5)
O3B	0.0160 (5)	0.0160 (5)	0.0307 (6)	0.0002 (4)	−0.0041 (5)	0.0065 (5)
O4B	0.0198 (5)	0.0349 (7)	0.0176 (5)	0.0002 (5)	−0.0015 (5)	0.0040 (5)
N1B	0.0133 (6)	0.0166 (6)	0.0177 (6)	0.0010 (5)	−0.0027 (5)	0.0017 (5)
N2B	0.0138 (6)	0.0281 (7)	0.0162 (6)	−0.0020 (5)	−0.0032 (5)	0.0013 (5)
C1B	0.0144 (6)	0.0181 (7)	0.0177 (6)	−0.0028 (5)	−0.0005 (5)	0.0007 (6)
C2B	0.0121 (6)	0.0238 (7)	0.0162 (6)	−0.0009 (6)	−0.0009 (5)	−0.0018 (6)
C3B	0.0125 (6)	0.0209 (7)	0.0176 (7)	0.0016 (6)	−0.0013 (5)	0.0001 (5)
C4B	0.0140 (6)	0.0164 (6)	0.0155 (6)	−0.0002 (5)	−0.0004 (5)	0.0007 (5)
C5B	0.0126 (6)	0.0178 (6)	0.0146 (6)	0.0001 (5)	−0.0011 (5)	−0.0002 (5)
C6B	0.0147 (6)	0.0169 (7)	0.0144 (6)	−0.0006 (5)	−0.0003 (5)	−0.0004 (5)
C7B	0.0158 (7)	0.0175 (7)	0.0194 (7)	−0.0010 (6)	−0.0023 (5)	0.0043 (5)
C8B	0.0158 (6)	0.0155 (6)	0.0201 (7)	−0.0001 (5)	−0.0021 (5)	0.0010 (6)
C9B	0.0118 (6)	0.0148 (6)	0.0173 (6)	0.0006 (5)	−0.0017 (5)	0.0018 (5)
C10B	0.0150 (7)	0.0227 (7)	0.0216 (7)	0.0006 (6)	−0.0025 (6)	−0.0044 (6)
C11B	0.0150 (7)	0.0269 (8)	0.0245 (8)	0.0022 (6)	−0.0050 (6)	−0.0047 (7)
C12B	0.0117 (6)	0.0214 (7)	0.0172 (6)	−0.0017 (5)	−0.0028 (5)	0.0022 (6)
C13B	0.0135 (6)	0.0211 (7)	0.0136 (6)	0.0000 (6)	0.0005 (5)	0.0020 (5)
C14B	0.0133 (6)	0.0196 (7)	0.0202 (7)	−0.0018 (6)	−0.0034 (5)	0.0028 (6)
C15B	0.0138 (7)	0.0227 (7)	0.0209 (7)	−0.0019 (6)	−0.0037 (6)	0.0020 (6)
C16B	0.0178 (8)	0.0448 (11)	0.0266 (8)	0.0049 (8)	−0.0022 (7)	−0.0105 (8)
C17B	0.0152 (8)	0.0567 (14)	0.0413 (11)	0.0071 (9)	−0.0048 (8)	−0.0138 (10)
C18B	0.0195 (8)	0.0418 (11)	0.0379 (10)	−0.0010 (8)	−0.0124 (8)	0.0021 (9)
C19B	0.0238 (9)	0.0661 (15)	0.0243 (8)	−0.0120 (10)	−0.0075 (7)	0.0066 (9)
C20B	0.0173 (8)	0.0568 (13)	0.0207 (8)	−0.0075 (8)	−0.0019 (6)	0.0036 (8)
C21B	0.0150 (7)	0.0342 (9)	0.0278 (8)	−0.0017 (7)	−0.0059 (6)	0.0069 (7)
C22B	0.0237 (8)	0.0167 (7)	0.0334 (9)	0.0044 (6)	−0.0083 (7)	0.0011 (6)

### Geometric parameters (Å, °)

**Table:**

O1A—C13A	1.233 (2)	O1B—C13B	1.2358 (19)
O2A—C2A	1.3708 (19)	O2B—C2B	1.3680 (19)
O2A—C21A	1.426 (2)	O2B—C21B	1.436 (2)
O3A—C4A	1.3706 (19)	O3B—C4B	1.3699 (19)
O3A—C22A	1.4352 (19)	O3B—C22B	1.4251 (19)
O4A—C14A	1.2342 (19)	O4B—C14B	1.235 (2)
N1A—C13A	1.348 (2)	N1B—C13B	1.346 (2)
N1A—C8A	1.464 (2)	N1B—C8B	1.460 (2)
N1A—C9A	1.479 (2)	N1B—C9B	1.4825 (19)
N2A—C14A	1.351 (2)	N2B—C14B	1.351 (2)
N2A—C12A	1.4507 (19)	N2B—C12B	1.4441 (19)
N2A—H2AA	0.8600	N2B—H2BA	0.8600
C1A—C2A	1.386 (2)	C1B—C6B	1.389 (2)
C1A—C6A	1.390 (2)	C1B—C2B	1.391 (2)
C1A—H1AA	0.9300	C1B—H1BA	0.9300
C2A—C3A	1.391 (2)	C2B—C3B	1.393 (2)
C3A—C4A	1.397 (2)	C3B—C4B	1.399 (2)
C3A—H3AA	0.9300	C3B—H3BA	0.9300
C4A—C5A	1.399 (2)	C4B—C5B	1.401 (2)
C5A—C6A	1.401 (2)	C5B—C6B	1.396 (2)
C5A—C9A	1.515 (2)	C5B—C9B	1.513 (2)
C6A—C7A	1.505 (2)	C6B—C7B	1.503 (2)
C7A—C8A	1.511 (2)	C7B—C8B	1.524 (2)
C7A—H7AA	0.9700	C7B—H7BA	0.9700
C7A—H7AB	0.9700	C7B—H7BB	0.9700
C8A—H8AA	0.9700	C8B—H8BA	0.9700
C8A—H8AB	0.9700	C8B—H8BB	0.9700
C9A—C10A	1.541 (2)	C9B—C10B	1.542 (2)
C9A—H9AA	0.9800	C9B—H9BA	0.9800
C10A—C11A	1.536 (2)	C10B—C11B	1.537 (2)
C10A—H10A	0.9700	C10B—H10C	0.9700
C10A—H10B	0.9700	C10B—H10D	0.9700
C11A—C12A	1.534 (2)	C11B—C12B	1.529 (2)
C11A—H11A	0.9700	C11B—H11C	0.9700
C11A—H11B	0.9700	C11B—H11D	0.9700
C12A—C13A	1.530 (2)	C12B—C13B	1.530 (2)
C12A—H12A	0.9800	C12B—H12B	0.9800
C14A—C15A	1.508 (2)	C14B—C15B	1.499 (2)
C15A—C20A	1.393 (2)	C15B—C16B	1.380 (2)
C15A—C16A	1.397 (2)	C15B—C20B	1.400 (2)
C16A—C17A	1.391 (2)	C16B—C17B	1.389 (3)
C16A—H16A	0.9300	C16B—H16B	0.9300
C17A—C18A	1.382 (3)	C17B—C18B	1.381 (3)
C17A—H17A	0.9300	C17B—H17B	0.9300
C18A—C19A	1.388 (2)	C18B—C19B	1.393 (3)
C18A—H18A	0.9300	C18B—H18B	0.9300
C19A—C20A	1.391 (2)	C19B—C20B	1.390 (2)
C19A—H19A	0.9300	C19B—H19B	0.9300
C20A—H20A	0.9300	C20B—H20B	0.9300
C21A—H21A	0.9600	C21B—H21D	0.9600
C21A—H21B	0.9600	C21B—H21G	0.9600
C21A—H21C	0.9600	C21B—H21E	0.9600
C22A—H22A	0.9600	C22B—H22G	0.9600
C22A—H22B	0.9600	C22B—H22D	0.9600
C22A—H22C	0.9600	C22B—H22E	0.9600
			
C2A—O2A—C21A	117.65 (15)	C2B—O2B—C21B	117.12 (13)
C4A—O3A—C22A	116.49 (13)	C4B—O3B—C22B	117.29 (13)
C13A—N1A—C8A	119.25 (14)	C13B—N1B—C8B	119.67 (13)
C13A—N1A—C9A	119.79 (13)	C13B—N1B—C9B	119.13 (13)
C8A—N1A—C9A	120.89 (13)	C8B—N1B—C9B	121.15 (12)
C14A—N2A—C12A	121.39 (13)	C14B—N2B—C12B	121.00 (13)
C14A—N2A—H2AA	119.3	C14B—N2B—H2BA	119.5
C12A—N2A—H2AA	119.3	C12B—N2B—H2BA	119.5
C2A—C1A—C6A	119.97 (15)	C6B—C1B—C2B	120.27 (15)
C2A—C1A—H1AA	120.0	C6B—C1B—H1BA	119.9
C6A—C1A—H1AA	120.0	C2B—C1B—H1BA	119.9
O2A—C2A—C1A	115.69 (15)	O2B—C2B—C1B	115.77 (14)
O2A—C2A—C3A	123.48 (16)	O2B—C2B—C3B	123.73 (14)
C1A—C2A—C3A	120.82 (15)	C1B—C2B—C3B	120.50 (14)
C2A—C3A—C4A	118.56 (16)	C2B—C3B—C4B	118.42 (14)
C2A—C3A—H3AA	120.7	C2B—C3B—H3BA	120.8
C4A—C3A—H3AA	120.7	C4B—C3B—H3BA	120.8
O3A—C4A—C3A	122.21 (15)	O3B—C4B—C3B	122.73 (14)
O3A—C4A—C5A	115.92 (14)	O3B—C4B—C5B	115.27 (13)
C3A—C4A—C5A	121.86 (15)	C3B—C4B—C5B	122.00 (14)
C4A—C5A—C6A	117.93 (15)	C6B—C5B—C4B	117.98 (14)
C4A—C5A—C9A	119.45 (14)	C6B—C5B—C9B	122.57 (13)
C6A—C5A—C9A	122.61 (15)	C4B—C5B—C9B	119.41 (14)
C1A—C6A—C5A	120.83 (16)	C1B—C6B—C5B	120.82 (14)
C1A—C6A—C7A	120.02 (15)	C1B—C6B—C7B	120.27 (14)
C5A—C6A—C7A	119.09 (15)	C5B—C6B—C7B	118.88 (13)
C6A—C7A—C8A	109.38 (13)	C6B—C7B—C8B	108.91 (12)
C6A—C7A—H7AA	109.8	C6B—C7B—H7BA	109.9
C8A—C7A—H7AA	109.8	C8B—C7B—H7BA	109.9
C6A—C7A—H7AB	109.8	C6B—C7B—H7BB	109.9
C8A—C7A—H7AB	109.8	C8B—C7B—H7BB	109.9
H7AA—C7A—H7AB	108.2	H7BA—C7B—H7BB	108.3
N1A—C8A—C7A	110.25 (14)	N1B—C8B—C7B	109.58 (13)
N1A—C8A—H8AA	109.6	N1B—C8B—H8BA	109.8
C7A—C8A—H8AA	109.6	C7B—C8B—H8BA	109.8
N1A—C8A—H8AB	109.6	N1B—C8B—H8BB	109.8
C7A—C8A—H8AB	109.6	C7B—C8B—H8BB	109.8
H8AA—C8A—H8AB	108.1	H8BA—C8B—H8BB	108.2
N1A—C9A—C5A	111.74 (13)	N1B—C9B—C5B	111.39 (12)
N1A—C9A—C10A	107.69 (12)	N1B—C9B—C10B	108.66 (12)
C5A—C9A—C10A	111.62 (13)	C5B—C9B—C10B	111.08 (12)
N1A—C9A—H9AA	108.6	N1B—C9B—H9BA	108.5
C5A—C9A—H9AA	108.6	C5B—C9B—H9BA	108.5
C10A—C9A—H9AA	108.6	C10B—C9B—H9BA	108.5
C11A—C10A—C9A	112.61 (13)	C11B—C10B—C9B	112.73 (13)
C11A—C10A—H10A	109.1	C11B—C10B—H10C	109.0
C9A—C10A—H10A	109.1	C9B—C10B—H10C	109.0
C11A—C10A—H10B	109.1	C11B—C10B—H10D	109.0
C9A—C10A—H10B	109.1	C9B—C10B—H10D	109.0
H10A—C10A—H10B	107.8	H10C—C10B—H10D	107.8
C12A—C11A—C10A	112.80 (13)	C12B—C11B—C10B	112.17 (13)
C12A—C11A—H11A	109.0	C12B—C11B—H11C	109.2
C10A—C11A—H11A	109.0	C10B—C11B—H11C	109.2
C12A—C11A—H11B	109.0	C12B—C11B—H11D	109.2
C10A—C11A—H11B	109.0	C10B—C11B—H11D	109.2
H11A—C11A—H11B	107.8	H11C—C11B—H11D	107.9
N2A—C12A—C13A	109.26 (13)	N2B—C12B—C11B	111.09 (13)
N2A—C12A—C11A	112.25 (13)	N2B—C12B—C13B	110.63 (13)
C13A—C12A—C11A	110.73 (13)	C11B—C12B—C13B	109.91 (13)
N2A—C12A—H12A	108.2	N2B—C12B—H12B	108.4
C13A—C12A—H12A	108.2	C11B—C12B—H12B	108.4
C11A—C12A—H12A	108.2	C13B—C12B—H12B	108.4
O1A—C13A—N1A	123.51 (15)	O1B—C13B—N1B	123.04 (15)
O1A—C13A—C12A	121.57 (14)	O1B—C13B—C12B	122.22 (14)
N1A—C13A—C12A	114.92 (14)	N1B—C13B—C12B	114.71 (13)
O4A—C14A—N2A	122.02 (15)	O4B—C14B—N2B	121.87 (14)
O4A—C14A—C15A	120.70 (14)	O4B—C14B—C15B	120.78 (15)
N2A—C14A—C15A	117.27 (13)	N2B—C14B—C15B	117.31 (14)
C20A—C15A—C16A	119.02 (15)	C16B—C15B—C20B	119.09 (16)
C20A—C15A—C14A	117.31 (13)	C16B—C15B—C14B	117.62 (15)
C16A—C15A—C14A	123.58 (15)	C20B—C15B—C14B	123.28 (16)
C17A—C16A—C15A	120.18 (16)	C15B—C16B—C17B	120.98 (17)
C17A—C16A—H16A	119.9	C15B—C16B—H16B	119.5
C15A—C16A—H16A	119.9	C17B—C16B—H16B	119.5
C18A—C17A—C16A	120.41 (16)	C18B—C17B—C16B	120.11 (19)
C18A—C17A—H17A	119.8	C18B—C17B—H17B	119.9
C16A—C17A—H17A	119.8	C16B—C17B—H17B	119.9
C17A—C18A—C19A	119.85 (16)	C17B—C18B—C19B	119.47 (18)
C17A—C18A—H18A	120.1	C17B—C18B—H18B	120.3
C19A—C18A—H18A	120.1	C19B—C18B—H18B	120.3
C18A—C19A—C20A	120.01 (16)	C20B—C19B—C18B	120.42 (18)
C18A—C19A—H19A	120.0	C20B—C19B—H19B	119.8
C20A—C19A—H19A	120.0	C18B—C19B—H19B	119.8
C19A—C20A—C15A	120.53 (15)	C19B—C20B—C15B	119.90 (18)
C19A—C20A—H20A	119.7	C19B—C20B—H20B	120.0
C15A—C20A—H20A	119.7	C15B—C20B—H20B	120.0
O2A—C21A—H21A	109.5	O2B—C21B—H21D	109.5
O2A—C21A—H21B	109.5	O2B—C21B—H21G	109.5
H21A—C21A—H21B	109.5	H21D—C21B—H21G	109.5
O2A—C21A—H21C	109.5	O2B—C21B—H21E	109.5
H21A—C21A—H21C	109.5	H21D—C21B—H21E	109.5
H21B—C21A—H21C	109.5	H21G—C21B—H21E	109.5
O3A—C22A—H22A	109.5	O3B—C22B—H22G	109.5
O3A—C22A—H22B	109.5	O3B—C22B—H22D	109.5
H22A—C22A—H22B	109.5	H22G—C22B—H22D	109.5
O3A—C22A—H22C	109.5	O3B—C22B—H22E	109.5
H22A—C22A—H22C	109.5	H22G—C22B—H22E	109.5
H22B—C22A—H22C	109.5	H22D—C22B—H22E	109.5
			
C21A—O2A—C2A—C1A	159.88 (17)	C21B—O2B—C2B—C1B	174.41 (15)
C21A—O2A—C2A—C3A	−21.3 (2)	C21B—O2B—C2B—C3B	−6.3 (2)
C6A—C1A—C2A—O2A	179.85 (15)	C6B—C1B—C2B—O2B	−179.61 (14)
C6A—C1A—C2A—C3A	1.0 (3)	C6B—C1B—C2B—C3B	1.0 (2)
O2A—C2A—C3A—C4A	−178.45 (15)	O2B—C2B—C3B—C4B	−179.22 (14)
C1A—C2A—C3A—C4A	0.3 (2)	C1B—C2B—C3B—C4B	0.1 (2)
C22A—O3A—C4A—C3A	−20.5 (2)	C22B—O3B—C4B—C3B	−16.2 (2)
C22A—O3A—C4A—C5A	160.26 (14)	C22B—O3B—C4B—C5B	164.01 (14)
C2A—C3A—C4A—O3A	178.87 (15)	C2B—C3B—C4B—O3B	179.13 (14)
C2A—C3A—C4A—C5A	−1.9 (2)	C2B—C3B—C4B—C5B	−1.1 (2)
O3A—C4A—C5A—C6A	−178.64 (14)	O3B—C4B—C5B—C6B	−179.22 (13)
C3A—C4A—C5A—C6A	2.1 (2)	C3B—C4B—C5B—C6B	1.0 (2)
O3A—C4A—C5A—C9A	0.1 (2)	O3B—C4B—C5B—C9B	−1.4 (2)
C3A—C4A—C5A—C9A	−179.18 (14)	C3B—C4B—C5B—C9B	178.84 (14)
C2A—C1A—C6A—C5A	−0.8 (3)	C2B—C1B—C6B—C5B	−1.2 (2)
C2A—C1A—C6A—C7A	176.35 (16)	C2B—C1B—C6B—C7B	176.58 (14)
C4A—C5A—C6A—C1A	−0.7 (2)	C4B—C5B—C6B—C1B	0.1 (2)
C9A—C5A—C6A—C1A	−179.43 (15)	C9B—C5B—C6B—C1B	−177.62 (14)
C4A—C5A—C6A—C7A	−177.87 (14)	C4B—C5B—C6B—C7B	−177.63 (13)
C9A—C5A—C6A—C7A	3.4 (2)	C9B—C5B—C6B—C7B	4.6 (2)
C1A—C6A—C7A—C8A	−143.01 (16)	C1B—C6B—C7B—C8B	−142.50 (14)
C5A—C6A—C7A—C8A	34.2 (2)	C5B—C6B—C7B—C8B	35.28 (19)
C13A—N1A—C8A—C7A	−136.11 (15)	C13B—N1B—C8B—C7B	−136.37 (14)
C9A—N1A—C8A—C7A	46.98 (19)	C9B—N1B—C8B—C7B	46.03 (18)
C6A—C7A—C8A—N1A	−57.11 (18)	C6B—C7B—C8B—N1B	−58.29 (16)
C13A—N1A—C9A—C5A	173.50 (13)	C13B—N1B—C9B—C5B	175.51 (12)
C8A—N1A—C9A—C5A	−9.60 (19)	C8B—N1B—C9B—C5B	−6.88 (19)
C13A—N1A—C9A—C10A	50.58 (18)	C13B—N1B—C9B—C10B	52.84 (17)
C8A—N1A—C9A—C10A	−132.52 (15)	C8B—N1B—C9B—C10B	−129.55 (14)
C4A—C5A—C9A—N1A	164.37 (13)	C6B—C5B—C9B—N1B	−20.2 (2)
C6A—C5A—C9A—N1A	−16.9 (2)	C4B—C5B—C9B—N1B	162.07 (13)
C4A—C5A—C9A—C10A	−74.98 (18)	C6B—C5B—C9B—C10B	101.07 (17)
C6A—C5A—C9A—C10A	103.70 (18)	C4B—C5B—C9B—C10B	−76.66 (17)
N1A—C9A—C10A—C11A	−52.65 (17)	N1B—C9B—C10B—C11B	−47.00 (18)
C5A—C9A—C10A—C11A	−175.65 (14)	C5B—C9B—C10B—C11B	−169.86 (14)
C9A—C10A—C11A—C12A	7.5 (2)	C9B—C10B—C11B—C12B	−2.2 (2)
C14A—N2A—C12A—C13A	−92.77 (17)	C14B—N2B—C12B—C11B	155.09 (16)
C14A—N2A—C12A—C11A	143.99 (16)	C14B—N2B—C12B—C13B	−82.56 (18)
C10A—C11A—C12A—N2A	164.68 (14)	C10B—C11B—C12B—N2B	172.47 (14)
C10A—C11A—C12A—C13A	42.28 (18)	C10B—C11B—C12B—C13B	49.70 (18)
C8A—N1A—C13A—O1A	4.1 (2)	C8B—N1B—C13B—O1B	0.6 (2)
C9A—N1A—C13A—O1A	−178.95 (14)	C9B—N1B—C13B—O1B	178.26 (14)
C8A—N1A—C13A—C12A	−176.20 (13)	C8B—N1B—C13B—C12B	178.49 (13)
C9A—N1A—C13A—C12A	0.7 (2)	C9B—N1B—C13B—C12B	−3.87 (19)
N2A—C12A—C13A—O1A	6.5 (2)	N2B—C12B—C13B—O1B	6.0 (2)
C11A—C12A—C13A—O1A	130.60 (15)	C11B—C12B—C13B—O1B	129.01 (16)
N2A—C12A—C13A—N1A	−173.23 (13)	N2B—C12B—C13B—N1B	−171.92 (12)
C11A—C12A—C13A—N1A	−49.10 (17)	C11B—C12B—C13B—N1B	−48.88 (17)
C12A—N2A—C14A—O4A	−17.9 (2)	C12B—N2B—C14B—O4B	−7.1 (3)
C12A—N2A—C14A—C15A	160.86 (14)	C12B—N2B—C14B—C15B	175.32 (15)
O4A—C14A—C15A—C20A	9.4 (2)	O4B—C14B—C15B—C16B	8.7 (3)
N2A—C14A—C15A—C20A	−169.43 (14)	N2B—C14B—C15B—C16B	−173.72 (18)
O4A—C14A—C15A—C16A	−174.28 (17)	O4B—C14B—C15B—C20B	−170.10 (19)
N2A—C14A—C15A—C16A	6.9 (2)	N2B—C14B—C15B—C20B	7.5 (3)
C20A—C15A—C16A—C17A	0.0 (3)	C20B—C15B—C16B—C17B	−1.3 (3)
C14A—C15A—C16A—C17A	−176.24 (17)	C14B—C15B—C16B—C17B	179.9 (2)
C15A—C16A—C17A—C18A	−0.3 (3)	C15B—C16B—C17B—C18B	0.3 (4)
C16A—C17A—C18A—C19A	0.3 (3)	C16B—C17B—C18B—C19B	1.1 (4)
C17A—C18A—C19A—C20A	0.0 (3)	C17B—C18B—C19B—C20B	−1.4 (4)
C18A—C19A—C20A—C15A	−0.3 (3)	C18B—C19B—C20B—C15B	0.4 (4)
C16A—C15A—C20A—C19A	0.2 (3)	C16B—C15B—C20B—C19B	1.0 (3)
C14A—C15A—C20A—C19A	176.76 (15)	C14B—C15B—C20B—C19B	179.7 (2)

### Hydrogen-bond geometry (Å, °)

**Table:**

*Cg*1 and *Cg*2 are the centroids of the C1A–C6A and C15A–C20A benzene rings, respectively.

**Table:**

*D*—H···*A*	*D*—H	H···*A*	*D*···*A*	*D*—H···*A*
N2A—H2AA···O1B^i^	0.86	2.08	2.8994 (18)	158
N2B—H2BA···O1A^ii^	0.86	2.24	2.9807 (18)	145
C1A—H1AA···O4B^iii^	0.93	2.52	3.405 (2)	160
C10A—H10B···O3A	0.97	2.58	3.127 (2)	115
C3B—H3BA···O4A^iv^	0.93	2.49	3.348 (2)	153
C7B—H7BB···O4A^v^	0.97	2.47	3.407 (2)	163
C16A—H16A···O1B^i^	0.93	2.32	3.228 (2)	165
C20B—H20B···O1A^ii^	0.93	2.51	3.281 (2)	141
C22A—H22A···O2A^iv^	0.96	2.52	3.266 (2)	135
C22A—H22B···O4B^vi^	0.96	2.47	3.393 (2)	160
C22B—H22D···O4A^iv^	0.96	2.58	3.414 (2)	146
C11A—H11B···Cg2^ii^	0.97	2.78	3.686 (2)	156
C8B—H8BA···Cg1^iv^	0.97	2.60	3.4864 (17)	152
C18B—H18B···Cg1^i^	0.93	2.81	3.607 (2)	144

Symmetry codes: (i) *x*−1/2, −*y*+3/2, −*z*; (ii) *x*+1/2, −*y*+3/2, −*z*; (iii) *x*, *y*+1, *z*;  (iv) −*x*+1, *y*−1/2, −*z*+1/2; (v) *x*+1, *y*, *z*; (vi) −*x*+1, *y*+1/2, −*z*+1/2.
